# Human-Induced Pluripotent Stem Cell-Derived Neural Stem Cell Therapy Limits Tissue Damage and Promotes Tissue Regeneration and Functional Recovery in a Pediatric Piglet Traumatic-Brain-Injury Model

**DOI:** 10.3390/biomedicines12081663

**Published:** 2024-07-25

**Authors:** Sarah L. Schantz, Sydney E. Sneed, Madison M. Fagan, Morgane E. Golan, Savannah R. Cheek, Holly A. Kinder, Kylee J. Duberstein, Erin E. Kaiser, Franklin D. West

**Affiliations:** 1 Regenerative Bioscience Center, University of Georgia, Athens, GA 30602, USA; sls46450@uga.edu (S.L.S.); sydney.sneed@uga.edu (S.E.S.); morgane.golan@uga.edu (M.E.G.); savannah.cheek@uga.edu (S.R.C.); hollyk17@uga.edu (H.A.K.); kyleejo@uga.edu (K.J.D.); 2 Biomedical and Health Sciences Institute, University of Georgia, Athens, GA 30602, USA; 3 Animal and Dairy Science Department, College of Agricultural and Environmental Sciences, University of Georgia, Athens, GA 30602, USA

**Keywords:** neural stem cells, TBI, porcine (pig) model, pediatrics, MRI, neurological evaluation, immunohistochemistry

## Abstract

Traumatic brain injury (TBI) is a leading cause of death and disability in pediatric patients and often results in delayed neural development and altered connectivity, leading to lifelong learning, memory, behavior, and motor function deficits. Induced pluripotent stem cell-derived neural stem cells (iNSCs) may serve as a novel multimodal therapeutic as iNSCs possess neuroprotective, regenerative, and cell-replacement capabilities post-TBI. In this study, we evaluated the effects of iNSC treatment on cellular, tissue, and functional recovery in a translational controlled cortical impact TBI piglet model. Five days post-craniectomy (n = 6) or TBI (n = 18), iNSCs (n = 7) or PBS (n = 11) were injected into perilesional brain tissue. Modified Rankin Scale (mRS) neurological evaluation, magnetic resonance imaging, and immunohistochemistry were performed over the 12-week study period. At 12-weeks post-transplantation, iNSCs showed long-term engraftment and differentiation into neurons, astrocytes, and oligodendrocytes. iNSC treatment enhanced endogenous neuroprotective and regenerative activities indicated by decreasing intracerebral immune responses, preserving endogenous neurons, and increasing neuroblast formation. These cellular changes corresponded with decreased hemispheric atrophy, midline shift, and lesion volume as well as the preservation of cerebral blood flow. iNSC treatment increased piglet survival and decreased mRS scores. The results of this study in a predictive pediatric large-animal pig model demonstrate that iNSC treatment is a robust multimodal therapeutic that has significant promise in potentially treating human pediatric TBI patients.

## 1. Introduction

Traumatic brain injury (TBI) is a leading cause of death and disability in the United States, with children under the age of four accounting for the largest population of TBI-related emergency department visits annually [1,2]. An estimated 2.3 million children, over 3% of all children in the US, have received a diagnosis of TBI. Considering nearly all neural connective development of sensory and language pathways, as well as a significant portion of higher cognitive function, occurs prior to 4 years of age, TBI-induced disruption of brain development can lead to lifelong learning, memory, motor function, and behavior deficits [3,4]. Despite a worldwide TBI epidemic, there is currently no Food and Drug Administration (FDA) approved treatment, and, therefore, a significant demand remains for novel therapeutics. Recent advancements in neural stem cell (NSC) therapy suggest the use of induced pluripotent stem cell-derived neural stem cells (iNSCs) are a promising therapeutic candidate due to their cell replacement, regenerative, and neuroprotective capabilities. Previous studies in rodent TBI models have demonstrated promising cellular and functional recovery following NSC treatment [5,6,7,8,9,10,11,12]. However, many treatments found to be highly efficacious in rodent models have failed in human clinical trials, likely due to inherent anatomical and physiological differences between rodents and humans. The failure to translate treatments from preclinical trials to the clinic has led to multiple calls for the testing of novel therapeutics in large animal models that have more comparable brain anatomy and physiology to humans [13,14,15]. Therefore, the use of a pediatric piglet TBI model, which is more representative of human pediatric patients in terms of brain size and neurodevelopmental processes, could help bridge the gap between preclinical rodent models and human clinical trials [16,17,18,19].

iNSCs are an exciting class of NSCs that can be used as an autologous NSC cell therapy, as they can be generated from the patient’s own body, with multimodal therapeutic mechanisms of action that have been shown to lead to cellular, tissue, and functional recovery in rodent TBI models [11]. Following TBI in rodents, NSCs have shown successful long-term survival and engraftment and replacement of damaged cells within lesioned tissues with proven capabilities of differentiating into neurons, astrocytes, and oligodendrocytes [6,7,9,12]. Furthermore, NSC-derived neurons showed neurite outgrowth, synapse formation, and electrical activity in rodent TBI models, indicating that NSC-derived mature cells are functional and capable of contributing to the recovery of neuronal activity [9,20]. Beyond cell replacement, NSCs have also shown proficient modulation of the cytotoxic TBI microenvironment through neuroprotective and regenerative paracrine signaling factors that promote tissue recovery [20]. NSCs produce neuroprotective and regenerative factors such as brain-derived neurotrophic factor (BDNF) and glial cell line-derived neurotrophic factor (GDNF), which are anti-apoptotic signaling factors that limit neural cell death [21,22] and stimulate endogenous tissue regeneration, resulting in increased numbers of Nestin+ endogenous NSCs and DCX+ neuroblasts [5,8]. Moreover, NSC treatment has been shown to mitigate neuroinflammation in TBI by reducing the recruitment of microglia and infiltrating macrophages and decreasing the expression of neural inflammatory cytokines and chemokines [5,6,8,22]. NSCs also produce blood–brain barrier stabilizing factors such as vascular endothelial growth factor (VEGF) and angiopoietin-1 (Ang1) that limit intracerebral hemorrhage and edema [21,23]. These same factors sustain vascular networks in the brain and promote angiogenesis, leading to improved cerebral blood flow (CBF). Furthermore, NSC-induced cellular and tissue level recovery has translated into increased cognitive performance in functional tests such as the Morris water maze, elevated plus maze, and radial arm maze, as well as enhanced motor recovery with improved performance in the rotarod and paw grasp tests for TBI animals [5,7,8,9,22].

Despite these promising results, iNSC therapeutic efficacy should be further validated in a translational large animal TBI model such as the pig, which possesses similarities in brain size, structural organization, development, myelination, vasculature, and inflammatory signature to that of humans [24]. Evaluation of iNSC treatment in the pig stroke model previously showed iNSCs enhanced neuron survival and reduced immune response, preserved white matter integrity and CBF, and decreased modified Rankin scores (mRS) post-stroke; however, similar treatment effects have yet to be validated in a piglet TBI model [21,25]. Furthermore, age is reported as one of the highest independent predictors of mortality and recovery following TBI [26]. However, most therapeutic intervention studies focus on adults and fail to acknowledge age-related differences in TBI sequelae. Simply evaluating the factor of size, the infant brain relative to the adult brain increases from 25% at birth to 95% by age 7. However, body weight increases from only 5% to about 30%, resulting in a disproportional weight distribution that leads to imbalance. This unbalanced weight distribution combined with the developing structure of the skull (e.g., incomplete closure of skull sutures) increases the likelihood of a fall and brain damage [27,28]. Furthermore, the developing brain possesses increased vulnerability compared to the mature brain as critical developmental activities such as neurogenesis, synaptogenesis, gliogenesis, and myelination peak before the age of four [28,29]. Due to known age-dependent differences related to neurotrophic development, programmed cell death, synaptic reorganization, neuroinflammation, and neurogenesis, researchers theorize the immature brain exhibits a unique response following TBI [30,31]. The pediatric population is, therefore, considered a vulnerable cohort, where physicians often inaccurately determine diagnosis and treatment protocols based on adult treatment paradigms. Young children commonly display unique delayed sequelae following injury, further complicating treatment protocols where unknown cognitive, social, and behavior deficits can remain hidden until years after the initial injury, likely due to an overlap between secondary injury progression and normal developmental periods [32,33]. Rodent TBI models have also revealed similar findings, with pediatric animals displaying a distinctive cell death time course and increased severity of tissue damage and degeneration [34,35,36]. These differences may be due to a unique age-specific immune response, where independent studies found juvenile mice to demonstrate an increased window of susceptibility to inflammatory stimuli relative to adults [37,38]. Furthermore, these models revealed worse functional outcomes, including decreased cognitive memory based on novel object testing and increased mortality [34,39]. While historically limited, the handful of studies discussed here highlight the imperative need to evaluate the efficacy of TBI therapeutics in an age-specific manner. Thus, the use of a piglet TBI model, age-matched in central nervous system development to a 2–3-year-old child, will enable a representative and robust evaluation of iNSC efficacy for pediatric TBI patients [40].

The objective of this study was to evaluate the potential of iNSCs to survive and differentiate in the brain parenchyma and to enhance cellular, tissue, and functional recovery in a translational pediatric piglet TBI model. For the first time, we demonstrated that perilesionally transplanted human iNSCs survived, differentiated, and integrated into post-TBI piglet brain tissue and enhanced endogenous neuroprotective and regenerative mechanisms. Tissue-level recovery identified through clinically relevant magnetic resonance imaging (MRI) revealed that iNSC therapy reduced tissue atrophy, lesion size, and consequential midline shift (MLS) while preserving CBF. Histological analysis further supported these results, showing that iNSC treatment led to increased neuronal and glial cell survival, decreased neuroinflammation, and augmented endogenous neurogenesis. Moreover, iNSC therapy improved neurological functional recovery and promoted the survivability of piglets following TBI. These results, therefore, support the continued investigation of iNSCs as a neuroprotective and regenerative therapy in follow-on pediatric TBI studies.

## 2. Materials and Methods

### 2.1. Animals and Housing

We used a total of twenty-four castrated male and intact female Yorkshire crossbreed 1-month-old piglets (N = 11, N = 13, respectively) for this study. All piglets were maintained in an environmentally controlled room with a 12-h light/dark cycle, maintained at room temperature (27 °C), and originated from the same genetic lineage to minimize experimental variability related to husbandry. Piglets were provided with free access to water and fed standard pig starter I, II, and grower diets with diets changing as recommended by swine nutritionists as the animals’ metabolic demands changed over time. Additionally, all piglets received daily enrichment through human contact, toys, and/or treats. Experiments were conducted in accordance with the National Institutes of Health (NIH) Guide for the Care and Use of Laboratory Animals guidelines. The University of Georgia Institutional Animal Care and Use Committee reviewed and approved all procedures and protocols prior to the beginning of the study (Animal Use Protocol A2019 07-007-Y3-A23). Piglets were group housed in a Public Health Service (PHS) and Association for Assessment and Accreditation of Laboratory Animal Care (AAALAC)-approved facility.

### 2.2. Study Design

After weaning, at approximately 3 weeks of age, piglets were randomly assigned to either TBI + human iNSCs (treated; n = 7), TBI + phosphate-buffered saline (PBS) (untreated; n = 11), or craniectomy + PBS (sham; n = 6) experimental groups using a randomized block design to minimize day effects. The initial study design allocated 6 animals per experimental group. Due to high mortality levels, additional animals were included in the study to ensure that a robust statistical analysis could be performed for key endpoints. Piglets underwent a 1-week habituation period after weaning to reduce handling stress during future procedures. iNSC treatment or PBS control was administered 5 days post-surgery. MRI collection occurred 1 day post-surgery to determine transplantation depths. At 12 weeks post-iNSC or PBS transplantation, all experimental piglets underwent MRI collection followed by sacrifice for brain tissue collection and analysis. Researchers directly responsible for the administration of the treatment were blinded during the process; additionally, all data were anonymized and underwent blinded analysis.

### 2.3. Controlled Cortical Impact

Approximately 12–18 h prior to surgery, a transdermal fentanyl patch (25 mcg/h; Covetrus, Portland, ME, USA) was applied to each piglet for pain management, and ceftiofur crystalline free acid (1 mL/44 lbs, intramuscular [IM]; Zoetis, Parsippany-Troy Hills, NJ, USA) was administered for pre-operative antibiotics. Pre-surgery sedation was achieved using xylazine (2 mg/kg IM; VetOne, Boise, ID, USA), midazolam (0.2 mg/kg IM; Heritage, Eatontown, NJ, USA), ketamine (2 mg/kg; Dechra Vet Products, Overland Park, KS, USA), and inhalant isoflurane (3.0–5.0% in oxygen; VetOne). Prophylactic 2.0% lidocaine (0.5 mL; VetOne) was administered topically to laryngeal folds, and propofol (0.5 mL to effect intravenous [IV] Zoetis) was administered to facilitate intubation (4–6 mm endotracheal tubes; VetOne). Anesthesia was maintained with isoflurane (1.0–2.0% in oxygen), and piglets were allowed to spontaneously respirate. Vitals, including heart rate, respiration rate, blood pressure, spO2, and rectal temperature, were continuously monitored throughout surgery and maintained within normal parameters.

A 4 cm left-sided incision was made at the top of the cranium to expose the underlying skull using an aseptic sterile technique. Liposomal bupivacaine (2 mg/kg; Nocita, Elanco, Greenfield, IN, USA) was used to desensitize the surgical window via periosteal block. At the left anterior junction of the coronal and sagittal suture lines of the skull, a 20 mm craniectomy was performed to expose the underlying dura of the motor cortex. Piglets were then secured in a controlled cortical impact device custom-built for our procedures (University of Georgia Instrument Design and Fabrication Shop, Athens, GA, USA). TBI at the motor cortex region was induced with a 15 mm blunt impactor tip with the following parameters: 4 m/s velocity, 12 mm depth of depression, and 400 ms dwell time. Studies previously conducted by our laboratory determined these parameters generated a moderate–severe TBI [16,18]. Sham piglets received only a craniectomy with no TBI. After either TBI induction or sham procedures, the surgical site was flushed with sterile saline. A second dose of bupivacaine was topically administered as an incisional block prior to closing the tissue with surgical sutures (synthetic absorbable braided PLGA; VetOne).

Post-operatively, piglets were monitored continuously until extubated. Once returned to their pen, piglets were monitored every 15 min until vitals returned to normal, then every 4 h for 24 h, and twice daily thereafter.

### 2.4. iNSC Culture

iNSCs (HIP^TM^ hNSC BC1, GlobalStem^®^, Rockville, MD, USA) purchased from GlobalStem and previously characterized by our group (Ref Baker paper) and shown to express NSC markers Nestin and Sox1 and capable of differentiation into neurons, astrocytes, and oligodendrocytes were maintained on coated tissue culture plates in neural stem cell media [21,25]. A complete media change was performed every other day, and cells were enzymatically passage at 90% confluency iNSCs were labeled with 1,1′-dioctadecyl-3,3,3′,3′-tetramethylindotricarbocyanine iodide (DiR) at a concentration of 2 µg/mL diluted in PBS 3 days prior to transplantation. The DiR labeling protocol was followed as previously described [21,25]. Cells were re-plated on coated tissue-culture-treated plates at 7.0 × 10^6^ cells per 150 mm plate and maintained as described above until transplantation.

### 2.5. iNSC Transplantation

All piglets received a transcranial transplantation of either iNSC treatment or PBS 5 days after TBI or sham surgery. Piglets were anesthetized according to the aforementioned pre- and intra-operative anesthesia protocols. The surgery site was routinely prepped and aseptically reopened for iNSC or PBS transplantation into the brain parenchyma. Transplantation surgeries were performed utilizing a large animal stereotaxic frame (David Kopf Instruments, Tujunga, CA, USA) with modifications to suit piglets. A mounted quintessential stereotaxic injector (Stoelting Co., Wood Dale, IL, USA) was utilized to inject 1.0 × 10^7^ DiR-labeled iNSCs at a rate of 2 µL/minute to prevent backflow. Immediately prior to injection, approximately 80 µL of iNSCs suspended in PBS (Dulbecco’s Phosphate Buffered Saline; Cytiva HyClone, Logan, UT, USA) or 80 µL of PBS control was sterilely loaded into a glass micropipette syringe with a 26-gauge needle (Hamilton Company, Reno, NV, USA) and attached to the stereotaxic apparatus.

Transplantation depth was determined using T2Weighted (T2W) MRI sequences in the coronal and sagittal planes. The total depth was equally subdivided into three separate injection sites to span grey and white matter compartments. This permitted the injection of 3 boli, approximately 27 µL each, spanning inferior to superior regions relative to the core of the injury. The needle was retracted at a rate of 1 mm/minute to prevent backflow. At the completion of transplantation, the epidermis was apposed via surgical suture. Anesthesia was discontinued, and the piglets were extubated and returned to their pens upon recovery. Vitals were monitored every 15 min until their return to normal; thereafter, monitoring was continued every 4 h for 24 h and twice daily for the remainder of the experiment.

### 2.6. MRI Acquisition and Analysis

MRI was performed 1 day post-surgery and 12 weeks post-transplantation using a General Electric 32-channel fixed-site Discovery MR750 3.0 Tesla MRI magnet (GE Healthcare, Chicago, IL, USA) and an 8-channel knee coil or 16-channel large flex coil placed over the cranium with the piglet positioned in supine recumbency. Piglets were sedated and maintained under anesthesia as previously described for pre- and post-operative procedures. Multiplanar MRI sequences were acquired, including T2W and arterial spin labeling (ASL). Sequence analysis was conducted using OsiriX software (Version 12.5.2; Bernex, Switzerland) at default thresholds.

Hemispheric atrophy and lesion volumes were semi-automatically calculated utilizing axial T2W sequences. Specifically, the ipsilateral hemisphere was manually identified in each axial slice by trained and blinded analysts. Once all ipsilateral hemisphere areas were defined, OsiriX-generated volumes for the ipsilateral hemisphere were reported as cm^3^. Lesions, identified by hyperintense regions of interest (ROIs), were outlined as described above. OsiriX-generated lesion volumes were then automatically calculated and reported as cm^3^.

MLS calculation was performed by utilizing coronal T2W sequences. The distance from the natural midline along the anterior and posterior attachments of the falx cerebri to the septum pellucidum was measured in each coronal slice. At the exact midpoint of the length of the septum pellucidum, the distance to the ideal midline was measured. This distance was calculated as the midline shift and reported in mm.

ASL sequences were utilized to assess changes in CBF between the ipsilateral and contralateral hemispheres. Manual identification of ROIs in the ipsilateral and contralateral hemispheres was performed in each coronal slice by trained blinded analysts. OsiriX-generated mean CBF values for both the ipsilateral and contralateral hemispheres were then calculated. CBF was reported as a percent change (calculated by comparing the mean CBF values in the ipsilateral hemisphere to the contralateral hemisphere), wherein a percent closer to zero equated to CBF being more similar to sham piglets.

### 2.7. Neurological Assessment

Piglets were assessed by a blinded, trained rater and assigned an mRS score pre-surgery; 0–2, 2–6, 6–12, and 12–24 h and 2 days post-surgery; 0–2, 2–6, 6–12, 12–24 h, 2 days, and 1, 2, 3, 4, 8, and 12 weeks post-transplantation. The mRS scale ranges from 0 to 6, where an animal receiving a 0 score would be entirely normal with no neurologic symptoms, an animal receiving a 3 score would exhibit moderate disability (e.g., requires some assistance but able to walk independently), and an animal receiving a 6 score would be deceased (Table S1). At each assessment point, piglets were evaluated by trained scorers on categories of general symptoms, eating/drinking, motor function, bodily function, level of consciousness, and deceased. At the conclusion of the study, all mRS assessments were blinded and then analyzed by a trained blinded researcher to assign each piglet a score at each time point. Piglets were assigned an mRS score according to their worst degree of neurologic dysfunction. For example, if an animal required assistance to eat/drink (mRS 3) but they were also circling (mRS 5), a score of 5 was recorded for that time point. This pig-adapted neurological scale has been proven capable of capturing functional deficits and recovery in our previous peer-reviewed published study [25].

### 2.8. Brain Tissue Collection and Processing

All piglets were euthanized following the 12-week post-transplantation MRI via euthanasia solution (1 mL/10 lbs IV; Euthasol, Virbac, Westlake, TX, USA). Immediately after euthanasia, piglet brains were removed and processed according to their treatment group. Specifically, sham and TBI+PBS piglets’ brains were immediately immersed in 10% neutral buffered formalin for later immunohistochemical analysis. For TBI+iNSC-treated piglets, the whole brain and coronal sections were imaged using Newton FT500 (18.11e; Vilber, Collégien, France). DiR fluorescence was visualized with the following acquisition parameters set in the Newton imaging interface: 740 nm excitation, F-800 filter, 100 ms exposure, 0.7 aperture, and super sensitivity. Consecutive 3 mm thick, coronal sections of the highest DiR expressing slices were collected. Slices were fixed in 4% paraformaldehyde (PFA, Electron Microscopy Sciences, Hatfield, PA, USA) for 24 h, dehydrated in 30% sucrose for 2 weeks, then embedded in Tissue-Tek OCT compound (Sakura, Torrance, CA, USA) and stored at −80 °C. The remaining brain sections were immersed in 10% buffered formalin to be preserved for immunohistochemistry (IHC) analysis.

### 2.9. Immunohistochemistry

IHC was performed as previously described by our laboratory [25]. Briefly, NeuN, Iba1, GFAP, and Olig2 images were taken along the lesion border (LB) of the whole ipsilateral hemisphere. For DCX, three separate anatomical regions were analyzed at the level of the caudate nucleus: the ventricular subventricular zone (vSVZ), the abventricular SVZ (aSVZ), and the LB. NeuN-, Olig2-, and DCX-positive cells were semi-automatically quantified using ImageJ 2.0 software (version 1.53k; Bethesda, MD, USA) and expressed as cells/mm^2^. Iba1 and GFAP were also semi-automatically quantified, where the total area of immunoreactivity corresponding to increased optical density was determined by ImageJ and expressed as a percent positive area.

### 2.10. Immunofluorescent Staining

OCT-embedded, fixed tissues were cryosectioned into 10–15 µm sections and mounted onto 76 × 127 mm gelatin-subbed glass slides. Slide-mounted tissue sections were stored at −20 °C. Immunofluorescent staining was performed as previously described, with a list of the antibodies provided in Table 1 [25]. Representative images were collected on a Cytation 5 reader (Biotek Instruments, Inc., Winooski, VT, USA).

### 2.11. Statistical Analysis

MRI and IHC quantitative data were statistically analyzed using the SAS software (version 9.3; Cary, NC, USA). Statistical significances between groups were determined by one-way ANOVA and post-hoc Tukey–Kramer Pair–Wise comparisons. The model included fixed effects for treatment and time, a treatment-by-time interaction, and a random intercept for each piglet, which was included to account for within-piglet correlation. mRS quantitative data were analyzed with GraphPad Prism (Version 10.2.2; San Diego, CA, USA). Statistical significances between groups and across time points were determined by two-way Mixed Model ANOVA and post-hoc Tukey–Kramer Pair–Wise comparisons. For all reported statistics, *p*-values ≤ 0.05 were considered significantly different, and data are reported as mean ± standard errors of the mean (SEM).

## 3. Results

### 3.1. Transplanted iNSCs Survived Long-Term and Differentiated into Neurons, Astrocytes, and Oligodendrocytes

**Figure 1:**
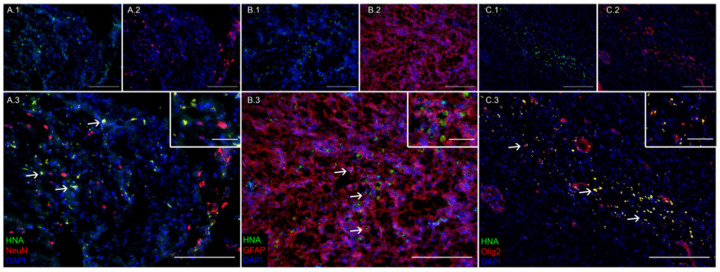
Transplanted iNSCs survived long-term and differentiated into neurons, astrocytes, and oligodendrocytes. Fluorescent immunohistochemistry performed 12 weeks post-transplantation revealed that HNA+ iNSC-derived cells survived and differentiated into the three major neural cell types: NeuN+ neurons (**A**), GFAP+ astrocytes (**B**), and Olig2+ oligodendrocytes (**C**), as determined by co-localization of HNA with the prior neural markers (white arrows). Original scale bars 200 µm, insert scale bars 50 µm.

Fluorescent IHC analysis of iNSC-treated TBI piglet brains 12 weeks post-transplantation showed iNSCs were capable of long-term survival, engraftment, and differentiation into mature neural cell types. Transplanted human iNSCs were identified by expression of the human nuclear antigen (HNA; Figure 1(A.1,B.1,C.1)). HNA+ cells were co-labeled with either NeuN (Figure 1(A.2)), GFAP (Figure 1(B.2)), or Olig2 (Figure 1(C.2)) to identify transplanted cells that differentiated into neurons (Figure 1(A.3)), astrocytes (Figure 1(B.3)), or oligodendrocytes (Figure 1(C.3)), respectively.

### 3.2. iNSC Treatment Led to Improved Neuron and Oligodendrocyte Survival and a Reduction in Immune Response 12 Weeks Post-Transplantation

**Figure 2:**
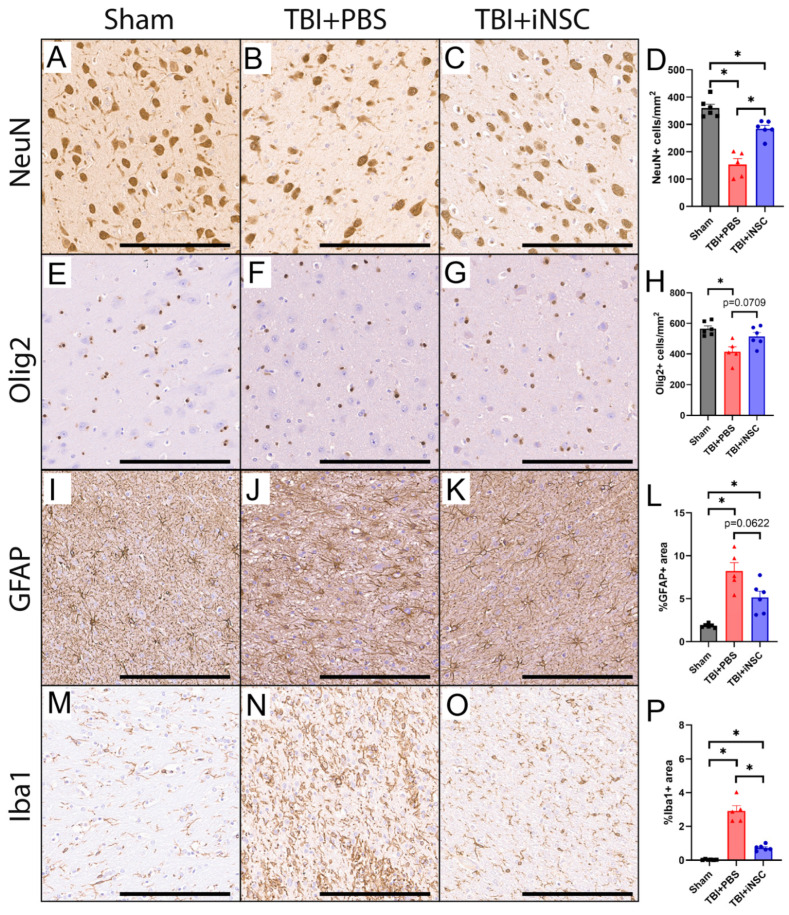
iNSC transplantation led to neuroprotection and amelioration of immunoreactivity. TBI+iNSC piglets showed significantly increased NeuN+ neurons at 12 weeks post-transplantation relative to TBI+PBS piglets (284.53 ± 12.29 vs. 154.10 ± 20.90 cells/mm^2^, respectively; (**A**–**D**). Olig2+ staining revealed a significant decrease in oligodendrocytes in TBI+PBS piglets but not TBI+iNSC piglets compared to shams (414.38 ± 32.32 vs. 514.43 ± 25.60 vs. 565.44 ± 18.18 cells/mm^2^, respectively; (**E**–**H**). GFAP+ staining indicated TBI+PBS piglets had a trending increase in the number of reactive astrocytes compared to TBI+iNSC piglets, while both TBI groups demonstrated increased GFAP+ staining compared to shams (8.22 ± 0.99 vs. 5.15 ± 0.72 vs. 1.86 ± 0.09%, respectively; (**I**–**L**). TBI piglets had significantly elevated levels of Iba1+ immune cells as compared to sham piglets (2.92 ± 0.32 vs. 0.73 ± 0.07 vs. 0.03 ± 0.01%, respectively; (**M**–**P**). However, TBI+iNSC piglets showed a significant reduction in Iba1+ immune cells relative to TBI+PBS piglets. * indicates a significant (*p* ≤ 0.05) difference between experimental groups. Data are expressed as mean ± SEM. Scale bars 200 µm.

The effect of iNSC treatment on endogenous neuron and oligodendrocyte survival and immune response was evaluated 12 weeks post-transplantation. TBI+iNSC piglets showed significantly greater numbers of NeuN+ neurons than TBI+PBS piglets (284.53 ± 12.29 vs. 154.10 ± 20.90 cells/mm^2^, respectively; Figure 2B–D), but still fewer than sham piglets (360.00 ± 13.98 cells/mm^2^; Figure 2A,D). Olig2 staining revealed a significantly decreased oligodendrocyte population in TBI+PBS piglets but not in TBI+iNSC treated piglets compared to sham piglets (414.38 ± 32.32 and 514.43 ± 25.60 vs 565.44 ± 18.18 cells/mm^2^, respectively; Figure 2E–H). Collectively, these results indicated that iNSC treatment led to the preservation of both neurons and oligodendrocytes at the lesion border as compared to PBS controls. Furthermore, evaluation of GFAP+ staining at the lesion border provided evidence that both TBI+PBS and TBI+iNSC piglets possessed a significant increase in reactive astrocytes at the lesion border compared to sham piglets (8.22 ± 0.99 and 5.15 ± 0.72 vs. 1.86 ± 0.09%, respectively; Figure 2I–L), yet TBI+iNSC piglets showed a trending decrease in GFAP+ staining relative to TBI+PBS piglets. A similar trend was observed in the number of Iba1+ immune cells as sham piglets demonstrated minimal positive staining (0.03 ± 0.01%; Figure 2M,P). However, TBI+iNSC piglets exhibited significantly decreased levels of Iba1+ cells as compared to TBI+PBS piglets (0.73 ± 0.07 vs. 2.92 ± 0.32%, respectively; Figure 2N–P). Statistical significances between groups were determined by one-way ANOVA and post-hoc Tukey–Kramer Pair–Wise comparisons.

### 3.3. iNSC Treatment Significantly Increased Neurogenesis Compared to Untreated Piglets

**Figure 3:**
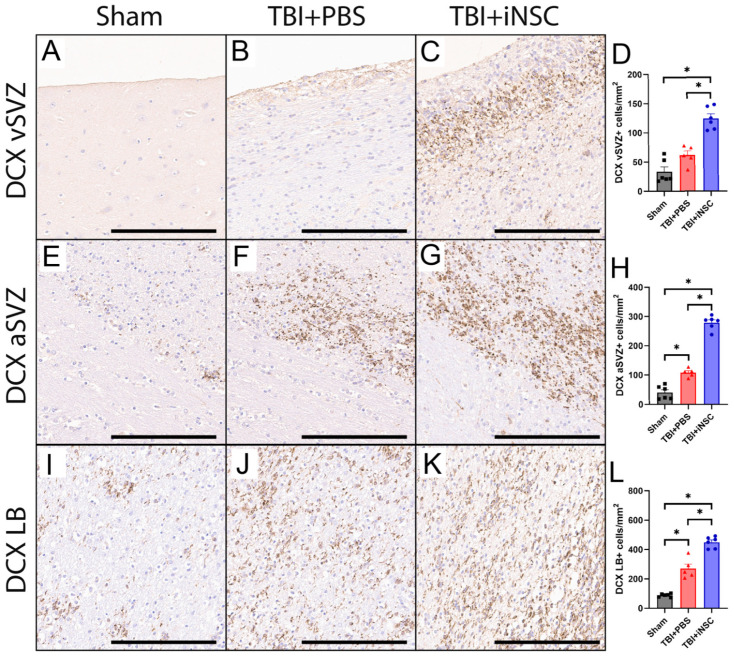
iNSC transplantation enhanced neuroblast proliferation. TBI+iNSC piglets showed a significant increase in DCX+ neuroblasts as compared to both TBI+PBS and sham piglets in the vSVZ (124.46 ± 7.74 vs. 61.59 ± 7.37 vs. 33.48 ± 8.19 cells/mm^2^, respectively; (**A**–**D**)), aSVZ (279.23 ± 9.57 vs.108.32 ± 6.61 vs. 40.39 ± 9.40 cells/mm^2^, respectively; (**E**–**H**)), and LB (449.17 ± 15.64 vs. 270.39 ± 30.27 vs. 88.33 ± 4.75 cells/mm^2^, respectively; (**I**–**L**)). * indicates a significant (*p* ≤ 0.05) difference between experimental groups. Data are expressed as mean ± SEM. Scale bars 200 µm.

Following a TBI, endogenous NSCs located in the SVZ can differentiate into neuroblasts, which migrate toward the LB to release neuroprotective neurotrophic factors and serve as a source for cell replacement. IHC analyses demonstrated TBI+iNSC piglets had significantly increased numbers of DCX+ neuroblasts compared to TBI+PBS and sham piglets in the vSVZ (124.46 ± 7.74 vs. 61.59 ± 7.37 and 33.48 ± 8.19 cells/mm^2^; Figure 3A–D), the aSVZ (279.23 ± 9.57 vs. 108.32 ± 6.61 vs. 40.39 ± 9.40 cells/mm^2^; Figure 3E–H), and the LB (449.17 ± 15.64 vs. 270.39 ± 30.27 vs. 88.33 ± 4.75 cells/mm^2^; Figure 3I–L). These results indicate that TBI+iNSC-treated piglets had a significant increase in neurogenesis, which resulted in increased numbers of neuroblasts in each region as compared to nontreated TBI piglets. TBI+PBS piglets also showed an increase in DCX+ neuroblasts compared to sham piglets in the aSVZ (108.32 ± 6.61 vs. 40.39 ± 9.40 cells/mm^2^) and LB (270.39 ± 30.27 vs. 88.33 ± 4.75 cells/mm^2^) as this neurogenic activation is a natural injury response to TBI. Statistical significances between groups were determined by one-way ANOVA and post-hoc Tukey–Kramer Pair–Wise comparisons.

### 3.4. iNSC Transplantation Significantly Decreased Ipsilateral Atrophy, Lesion Volume, and Midline Shift While Preserving Cerebral Blood Flow

**Figure 4:**
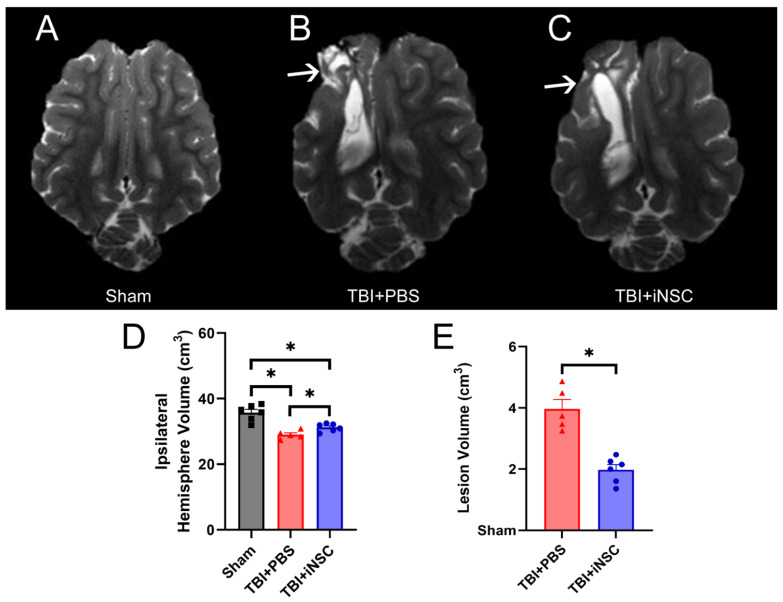
iNSC transplantation decreased tissue atrophy and lesion volume. Axial T2W sequences for sham (**A**), TBI+PBS (**B**), and TBI+iNSC (**C**) piglets revealed a significant decrease in ipsilateral hemisphere volume between sham and TBI piglets (35.73 ± 0.10 vs. 29.01 ± 0.57 vs. 31.20 ± 0.49 cm^3^, respectively; (**D**)). Interestingly, TBI+PBS piglets also demonstrated a significant decrease in hemisphere volume compared to TBI+iNSC piglets, thus indicating increased atrophy in TBI+PBS piglets. Hyperintense lesion volumes (white arrows, (**B**,**C**)) were significantly decreased in TBI+iNSC piglets as compared to TBI+PBS piglets (1.98 ± 0.17 vs. 3.97 ± 0.31 cm^3^, respectively; (**E**)). * indicates a significant (*p* ≤ 0.05) difference between experimental groups. Data are expressed as mean ± SEM.

Clinically relevant MRI sequences assessed tissue-level recovery in iNSC-treated TBI piglets as compared to PBS control TBI piglets 12 weeks post-transplantation. T2W sequences (Figure 4A–C) revealed a significant decrease in ipsilateral hemisphere volumes in TBI piglets compared to sham piglets. However, TBI+iNSC piglets exhibited significantly larger ipsilateral hemisphere volumes compared to TBI+PBS piglets (29.01 ± 0.57 and 31.20 ± 0.49 vs. 35.73 ± 0.10 cm^3^, respectively; Figure 4D), thus suggesting iNSC treatment decreased tissue atrophy. Hyperintense lesion volumes (Figure 4B,C, white arrows) were also decreased in TBI+iNSC piglets compared to TBI+PBS piglets 12 weeks post-transplantation (1.98 ± 0.17 vs. 3.97 ± 0.31, respectively; Figure 4E).

**Figure 5:**
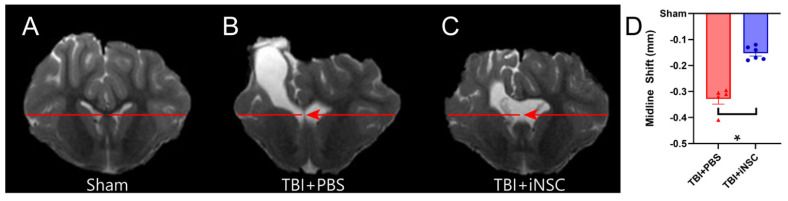
iNSC transplantation decreased midline shift. Coronal T2W sequences for sham (**A**), TBI+PBS (**B**), and TBI+iNSC (**C**) piglets revealed a significant reduction in MLS (red arrows) towards the ipsilateral hemisphere in TBI+iNSC piglets compared to TBI+PBS piglets (−0.33 ± 0.02 vs. −0.15 ± 0.01 mm, respectively; (**D**)). * indicates a significant (*p* ≤ 0.05) difference between experimental groups. Data are expressed as mean ± SEM.

**Figure 6:**
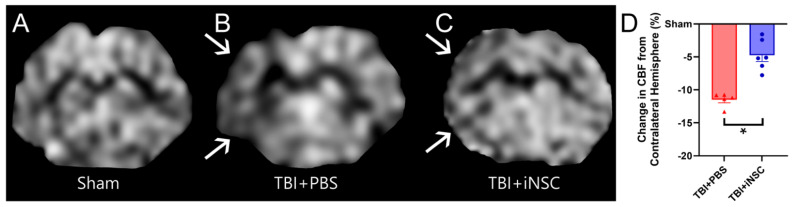
iNSC transplantation preserved cerebral blood flow. ASL-generated CBF maps for sham (**A**), TBI+PBS (**B**), and TBI+iNSC (**C**) piglets showed that TBI+PBS piglets possessed a greater decrease in CBF (hypointense regions, white arrows) than TBI+iNSC piglets (−11.48 ± 0.47 vs. −4.73 ± 0.96%, respectively; (**D**)). * indicates a significant (*p* ≤ 0.05) difference between experimental groups. Data are expressed as mean ± SEM.

Greater midline shifts have long been associated with increased mortality and morbidity, while lower shifts have been used to predict favorable recovery outcomes post-neurological injury [41]. TBI+iNSC piglets showed a significant reduction in MLS (Figure 5A–C; red lines) towards the affected ipsilateral hemisphere compared to TBI+PBS piglets (−0.15 ± 0.01 mm vs. −0.33 ± 0.02 mm respectively; Figure 5B). Alterations in CBF following TBI have been linked to underlying functional disability and found to precede TBI-induced neurodegeneration following injury [42]. ASL (Figure 6A–C) revealed TBI+iNSC piglets exhibited a lower percent decrease in CBF (white arrows) compared to TBI+PBS piglets (−4.73 ± 0.96 vs. −11.48 ± 0.47%, respectively; Figure 6D). Statistical significances between groups were determined by one-way ANOVA and post-hoc Tukey–Kramer Pair–Wise comparisons.

### 3.5. Treatment with iNSCs Improved Neurological Performance and Survivability Post-TBI

**Figure 7:**
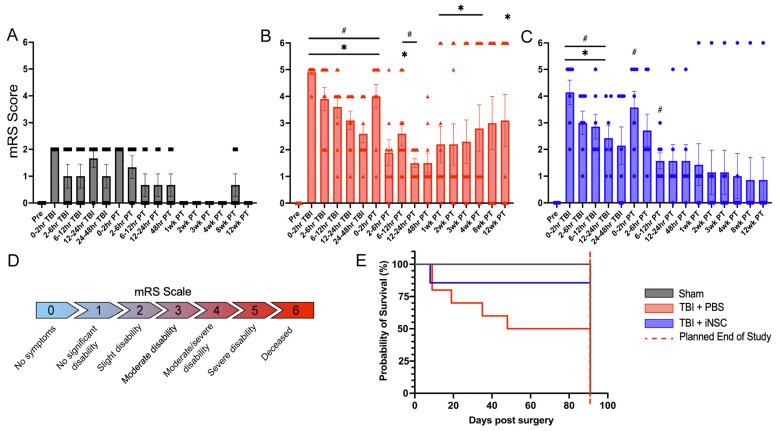
iNSC transplantation decreased neurological disability and increased piglet survivability at acute and chronic time points. A mRS scale (**D**) was used to assess neurological disability with scores ranging from 0 (healthy) to 6 (death). The mean mRS scores for the sham (**A**), TBI+PBS (**B**), and TBI+iNSC (**C**) animals are represented as either pre-surgery (Pre), post-surgery [Craniectomy (Cran) or TBI], or post-transplantation (PT). TBI+PBS animals exhibited significantly greater deficits compared to sham piglets from 0–48 h (h) post-TBI and at 6–12 h PT and 1, 2, 3, 4, and 12 weeks (wk) PT. TBI+iNSC treated animals displayed significantly greater deficits compared to sham animals only from 0–12 hr post-TBI, with no differences in functional deficits observed PT. Additionally, TBI+PBS piglets displayed significantly increased deficits compared to their baseline score through 24 hr PT, compared to TBI+iNSC, which did not display significantly increased deficits after 12 h PT. iNSC-treated piglets exhibited an 85.7% chance of survival, whereas PBS control piglets exhibited only a 50% chance of survival at the conclusion of the study (87.17 ± 13.83 vs. 57.50 ± 11.74 average days survived after surgery, respectively; (**E**)). * indicates significant (*p* ≤ 0.05) difference compared to sham at the same timepoint; # (*p* ≤ 0.05) indicates significant difference compared to pre-surgery.

The mRS scale was used to evaluate neurological disability post-surgery. Scores ranged from a normal score of 0 to a moderately disabled score of 3 to a score of 6 for deceased piglets. Prior to transplantation, mRS scores revealed moderate to severe functional deficits in both TBI experimental groups. These piglets required consistent critical care due to neurological deficits. Post-transplantation, TBI+PBS piglets continued to demonstrate significantly increased mRS scores compared to sham piglets at 0–2 h (4.00 ± 0.45 vs. 2.00 ± 0.00), 1 week (2.20 ± 0.68 vs. 0.00 ± 0.00), 2 weeks (2.20 ± 0.77 vs. 0.00 ± 0.00), 3 weeks (2.30 ± 0.82 vs. 0.00 ± 0.00), 4 weeks (2.80 ± 0.88 vs 0.00 ± 0.00), and 12 weeks (3.10 ± 0.97 vs. 0.00 ± 0.00; Figure 7A,B). In contrast, TBI+iNSC-treated piglets did not demonstrate increased functional deficits relative to sham piglets following iNSC transplantation through the remainder of the study (Figure 7C). Within treatment groups, TBI+PBS animals showed significantly greater deficits post-transplantation relative to their pre-surgery baseline score from 0–2 h (4.00 ± 0.45 vs. 0.00 ± 0.00), 6–12 h (2.60 ± 0.45 vs. 0.00), and 12–24 h (1.50 ± 0.17 vs. 0.00). Conversely, TBI+iNSC piglets demonstrated significantly greater deficits post-transplantation relative to their pre-surgery baseline score at only 0–2 h (3.57 ± 0.61 vs. 0.00 ± 0.00) and 6–12 h (1.57 ± 0.30 vs. 0.00 ± 0.00). Furthermore, TBI+iNSC piglets exhibited an 85.7% chance of survival, whereas TBI+PBS piglets exhibited only a 50.0% chance of survival (87.17 ± 13.83 vs. 57.50 ± 11.74 average days survived after surgery, respectively; Figure 7D). Specifically, 1 TBI+iNSC piglet and 4 TBI+PBS piglets were euthanized prior to the planned end of the study upon reaching predetermined IACUC-approved humane endpoints. Specific necropsy details for each animal can be found in Table S2. Overall, these differences between the TBI+PBS and TBI+iNSC piglets post-transplantation suggest that iNSC treatment supports faster functional recovery and improves survivability post-TBI. Statistical significances between groups and across time points were determined by two-way Mixed Model ANOVA and post-hoc Tukey–Kramer Pair–Wise comparisons.

## 4. Discussion

iNSC treatment possesses significant potential as a novel TBI therapy due to its multimodal cellular replacement, neuroprotective, and regenerative capacity [5,6,7,8,9,10,11,12,22]. Successful preclinical studies in rodent models indicate NSC treatment induces substantial recovery following injury [11,43,44,45]. However, until now, NSC treatment had yet to be tested in a preclinical large animal TBI model that has a more comparable neuroanatomy and TBI pathophysiology to humans and is therefore likely to be more predictive of human NSC therapeutic responses, an important and repeatedly recognized de-risking step as TBI therapeutics transition from bench to bedside [13,14,15]. Furthermore, the therapeutic capacity of NSCs in a pediatric animal model had yet to be fully examined. By addressing this knowledge gap in a pediatric TBI piglet model, we provide the first experimental evidence that iNSCs are capable of long-term engraftment and differentiation into neurons, astrocytes, and oligodendrocytes in the developing piglet brain post-TBI. iNSC treatment was neuroprotective, immunomodulatory, and regenerative, as demonstrated by increased survival of endogenous neurons and oligodendrocytes, reduced immune cell activity, and enhanced neurogenesis in the TBI brain. These cellular-level improvements corresponded with decreased hemispheric atrophy, MLS, and lesion volume while also preserving CBF within the ipsilateral hemisphere. iNSC therapy reduced functional deficits at acute and chronic time points as assessed by the mRS neurological disability scale and increased survivability rates in piglets. Together, these findings demonstrate that transplantation of iNSCs promotes significant cellular, tissue, and functional recovery, thus making it a potentially transformative treatment option for pediatric TBI patients.

In this study, the neuroprotective effect of iNSC treatment led to significant preservation of ipsilateral hemisphere volumes and reduced lesion volumes in TBI piglets, thus showing reduced tissue atrophy and supporting our histological results demonstrating preservation of neurons and oligodendrocytes at the cellular level. iNSC-treated piglets also possessed a decreased MLS at 12 weeks post-transplantation when compared to PBS controls, again supporting tissue preservation. In pediatric TBI patients, volumetric measurements of tissue atrophy, lesion size, and consequential MLS are highly correlated with survivability and functional recovery [46]. Rodent studies evaluating the effect of NSC treatment, however, observe mixed efficacy in relation to TBI lesion volume and generally do not evaluate hemispheric atrophy or MLS [5,6,7,9,47,48]. Interestingly, an evaluation of recent rodent studies assessing the effect of NSC treatment in TBI [5,6,7,9,47,48] showed that 50% of the studies demonstrated a significant reduction in TBI lesion volume [9,47,48], while the remaining 50% reported no statistical differences [5,6,7]. In contrast to our results, Badner et al. found that NSCs transplanted 30 days post-injury had no effect on lesion volume and had limited effects on hippocampal neuron preservation, yet reduced neuroinflammation and significantly improved performance on the elevated plus maze in rats [5]. Similarly, Haus et al. demonstrated that NSC transplantation 9 days post-injury had no effect on lesion volume or spared tissue volume yet showed a significant increase in hippocampal neuron survival that corresponded with reduced long-term cognitive deficits in rats [7]. The authors of both studies suggested transplantation may have been outside the optimum window of tissue-damage mitigation for a severe injury [5,7]. Therefore, the lesion likely stabilized following injury, and the functional recovery observed may be a result of NSC-mediated hippocampal neuroprotection. Additionally, Gao et al. showed NSC treatment led to a limited change in lesion volume and attributed the lack of differences between treated and non-treated rats to large variations in severe CCI [6]. In contrast, Lin et al. found that NSC treatment in TBI mice led to comparable results to those found in this study [9]. Specifically, Lin et al. demonstrated that NSC treatment led to a significant reduction in lesion volume that corresponded with successful NSC neuronal differentiation and integration into damaged tissues. NSC TBI treatment also correlated with improved cognitive and motor function performance as evaluated by the Morris water maze and rotarod tasks. These results suggest that NSC treatment can function as a cell replacement and neuroprotective therapy that can reduce lesion volume and improve functional recovery. Hu et al. similarly demonstrated lesion volume reduction with corresponding motor-function improvements [48]. Interestingly, Hu et al. further revealed a location-dependent effect of transplantation site on lesion volume where NSCs delivered perilesionally significantly reduced lesion volume, while intralesional NSC transplantation did not lead to lesion reduction. Moreover, perilesional NSC transplantation increased cortical tissue sparing compared to intralesional NSC transplantation. However, no differences in NSC engraftment were found, thus suggesting that NSC-dependent lesion volume reduction is due to neuroprotective and/or regenerative mechanisms of action rather than cellular replacement [48]. These results support the findings of our study that showed decreased lesion volume corresponds with significant neuronal and oligodendrocyte sparing and reduced neurological deficits with relatively low engraftment levels, indicating an underlying neuroprotective signaling mechanism in mitigating lesion volume rather than cellular replacement. There are several differences that may explain the observed inconsistencies between studies in NSC prevention of brain lesioning, including differences in NSC origin, dosage, administration window, and transplant location which are all highly variable between studies. For example, Narouiepour et al. [10] showed that 5 × 10^5^ fetal-derived NSCs delivered transcranially 10 min post-TBI produced no effect on lesion volume, while Lin et al. [9] showed delivery of 1 × 10^5^ embryonic-derived NSC transcranially 1 day post-TBI significantly decreased lesion volume despite being fewer cells and transplanted at a later timepoint post-TBI. In this study, we transplanted 1.0 × 10^7^ iNSCs perilesionally 5 days post-TBI and demonstrated a significant reduction in lesion volume and chronic tissue atrophy, spared endogenous cell populations, and reduced functional deficits. The high degree of variability in NSC therapeutic efficacy suggests that future studies are needed to further assess optimal administration protocols to produce more consistent results in NSC-mediated TBI recovery.

In addition to structural changes in the brain, TBI often leads to alterations in CBF indicative of neurovascular injury, which is correlated with aberrant neuronal activity and cognitive function [49,50,51]. Perturbations in CBF post-TBI are commonly used as a predictive metric due to strong correlations between decreased CBF and poor outcomes on the Pediatric Glasgow Outcome Scale Extended (GOSE-Peds) and Post-Concussion Symptom Inventory (PCSI) [49,52]. In this study, iNSC-treated piglets demonstrated a greater preservation of CBF within the ipsilateral hemisphere when compared to non-treated piglets, which corresponded with a reduction in neurological deficits based on mRS scores. However, targeting restoration of CBF within the pediatric brain has proven to be challenging as it is highly variable based on age due to the dynamic metabolic energy demands during specific developmental windows (e.g., CBF volume in neonates is 70 mL/min and about 700 mL/min in 3-year-old children), which results in uniquely challenging differences in injury responses [28,53,54,55]. The difficulty in evaluating CBF-related therapeutic responses in the pediatric brain highlights the value of evaluating CBF changes within our pediatric gyrencephalic piglet TBI model. The authors theorize the observed preservation of CBF in this study is likely due to the production of vascular stabilizing and angiogenic factors, such as VEGF and Ang1, from transplanted iNSCs [56]. Elevated expression of angiogenic factors has been dynamically related to increased endogenous angiogenesis and the preservation of CBF in both rodent and pig neural injury models [21,23,57].

Interestingly, microglia have been shown to be present in higher numbers in the developing brain and play a unique role in neurodevelopmental processes. Microglia provide early post-natal neurotrophic support through nerve growth factor (NGF) and BDNF expression and promote neuronal differentiation and white matter development [58,59,60]. However, this elevated microglia population required for normal healthy brain development can enhance the occurrence of inflammation and reactive oxygen species (ROS) formation in response to injury. The immature brain is less equipped to combat oxidative stress due to the reduced antioxidant capacity associated with decreased glutathione peroxidase activity levels relative to the adult brain [59,61,62]. Therefore, the pediatric brain is more susceptible to inflammatory and oxidative secondary injury in response to TBI. To further complicate, it remains difficult to fully understand the pathological heterogeneity observed in TBI patients as the secondary injury cascade is linked to complex molecular mechanisms that influence injury severity [63]. At acute TBI time points, both microglia, the endogenous immune cells of the brain, and circulating immune cells that infiltrate the injured brain are activated and promote inflammation via the production of cytokines and chemokines such as IL-1β and CXCR-4 [19,38,64,65,66]. Inflammation can chronically persist for months and even years post-TBI, leading to further neural cell death, axonal degeneration, and tissue atrophy which have been shown to exacerbate functional deficits [67]. Notably, in the present study, iNSC-treated piglets demonstrated a significantly reduced population of Iba1+ immune cells at the lesion border. This supports the longitudinal anti-inflammatory, neuroprotective effect of NSCs. Previous rodent TBI studies showed NSC treatment reduces acute inflammation through decreased recruitment of CD16+ and MHC11+ infiltrating pro-inflammatory immune cells and subsequent depression of pro-inflammatory cytokine expression (e.g., IFN-γRβ) and elevated anti-inflammatory cytokine expression (e.g., Il-4Ra) following TBI [8,22].

In the present study, we demonstrated a significant increase in neuroblast populations at the vSVZ, aSVZ, and LB in all TBI piglets relative to sham controls, supporting that TBI alone stimulates neurogenic activity, which is in agreement with previous studies [18,68,69]. The endogenous, neurogenic TBI repair mechanism responds to injury by promoting activation and amplification of NSCs, followed by NSC-derived neuroblasts migrating to the perilesional region. Neuroblasts then differentiate into mature neurons capable of integrating within the damaged neural tissue [68,69]. However, functional cellular replacement can be limited due to the lack of appropriate organizational cues that are typically present during normal development and the cytotoxic post-TBI environment, resulting in failed engraftment and improper cellular connectivity [69]. Furthermore, when experimentally evaluating neurogenesis at the SVZ, it is important to consider that differences in regenerative endogenous responses between studies are largely dependent on the animal model, injury severity, and time point [70]. For example, while neuroblast migration has been observed at acute time points in rodent TBI studies [68,69], Costine et al. reported limited neuroblast migration at similar time points in piglet TBI studies, even though piglets have similar vSVZ and aSVZ regions to that of children [71]. The authors suggested that increased time post-TBI is required to allow for such neuroblast migration in a larger-brained species [71]. Kinder and colleagues later identified increased populations of DCX+ neuroblasts in piglets 28 days post-TBI; however, long-term consequences of neuroblast migration were not evaluated [18]. In this study, iNSC treatment augmented neuroblast proliferation and migration as treated piglets possessed a significant increase in DCX+ neuroblasts in the vSVZ, aSVZ, and LB compared to non-treated piglets. This enhanced neuroblast proliferation and migration from the vSZV through the aSZV to the LB is likely due to the ability of iNSCs to upregulate neuroblast proliferative genes such as BDNF, VEGF, and Noggin (NOG), which have been found to increase in response to iNSC treatment in other preclinical studies [21,23,72]. While NSC-dependent neurogenesis is well documented within mature rodent TBI models, we are the first to provide evidence that iNSCs enhance neurogenesis following TBI in a pediatric gyrencephalic model to our knowledge, emphasizing again the importance of pediatric-specific models for therapeutic intervention [8,9,21,25].

Previous preclinical studies have shown strong evidence linking NSC treatment to increased survival and decreased functional deficits following TBI [8,9,10,73]. In support of this, iNSC-treated TBI piglets in this study demonstrated a survivability rate of 85.7%, whereas non-treated TBI piglets showed a decreased survivability rate of only 50.0%. Interestingly, the survivability of untreated TBI pigs in this study is highly comparable to severe human pediatric TBI survivability rates at 43% survivability [74]. iNSC treatment also reduced neurological deficits with mRS scores comparable to sham animals at acute and chronic time points post-transplantation, highlighting a significant improvement in functional outcomes corresponding with scores of <4. Similarly, the ability of NSC treatment to mitigate neurological and motor function deficits in rodent TBI models is well documented, with animals showing improvements in neurological severity scores (NSS) and Bederson neurological exam and motor function performance tasks including the elevated body swing test (EBST) and rotarod [8,9,10,73]. Lee et al. demonstrated high doses of NSCs significantly mitigated Benderson neurological scores and EBST performance compared to baseline, yet interestingly, found no treatment effects on rotarod performance [8]. The authors of this study proposed that higher doses of NSCs may further improve neurological scores and may be required to observe rescue of locomotor function. Of note, Lee et al. evaluated rotarod performance on days 7, 14, 30, 60, and 90 post-TBI [8], in contrast to Lin et al., which revealed significant improvements in rotarod performance at acute time points of days 1, 3, and 7 post-injury [9]. However, after day 14, Lin et al. similarly demonstrated no treatment effects, likely due to a lack of sensitivity of the rotarod task in repetitive evaluations of chronic motor function deficits [8,9]. Therefore, the ability to reveal both acute and chronic neurological deficits while accounting for motor function recovery following iNSC treatment highlights the strength of using mRS scores in this study. The use of the mRS scale in this study also increases the translational therapeutic potential of iNSCs, as the mRS scale is used in numerous clinical trials as an endpoint in TBI. Collectively, these findings suggest that iNSC treatment led to improved cellular and tissue recovery and ultimately enhanced survivability and neurological recovery.

The findings of this study show that iNSC treatment led to significant improvement in TBI recovery. However, these results must be interpreted while considering the limitations of this study, including low iNSC engraftment levels. Although iNSCs survived long-term and were capable of neuronal differentiation, the low quantity of HNA+ cells 12 weeks post-transplantation suggests that the majority of the observed iNSC therapeutic effects were due to neurotrophic factor signaling and not cellular replacement. The low level of iNSC engraftment supports the potential need for pre-transplantation treatment with additional therapeutics that can mitigate the cytotoxic TBI environment and promote iNSC engraftment. It is likely that increased iNSC survival and engraftment will further promote iNSC-derived neuroprotective and regenerative signaling and a global increase in recovery responses. Recent studies, such as Kaiser et al., have demonstrated pre-treatment with anti-inflammatory and antioxidative Tanshinone IIA-loaded nanoparticles prior to iNSC transplantation led to a 77% increase in iNSC engraftment in a porcine stroke model [25]. Studies in rodent TBI models have also shown improved engraftment by combining NSC transplantation with scaffold delivery, genetically modifying NSCs pre-transplantation, and joint NSC transplantation with drug therapy [75,76,77]. Follow-on studies evaluating an iNSC combination therapy in a translational pediatric piglet TBI model, therefore, present a promising opportunity to enhance NSC treatment effects.

## 5. Conclusions

For the first time, this study demonstrated in a translational piglet TBI model the potential for iNSC therapy to serve as a multimodal neuroprotective, immunomodulatory, and regenerative pediatric TBI treatment that promotes significant cellular, tissue, and functional recovery. Capable of long-term survival and differentiation into neural cell types, transplanted iNSCs increased neural cell survival and neurogenesis while mitigating immune responses. iNSC treatment ultimately led to reduced atrophy, lesion volumes, MLS, and increased CBF. Improvements in neurological performance and piglet survivability reinforce the need for further investigations that will continue to explore underlying mechanisms associated with iNSC efficacy. Preclinical investigations such as this study may aid in moving iNSCs to the clinic as a potential treatment option for a large and growing population of pediatric TBI patients that currently have no access to an FDA-approved therapy.

## Figures and Tables

**Table 1:** Antibody Information.

Target	Species	Dilution	Manufacturer
NEUN	Rabbit	1:750	Novus NBP1-77686 (Centennial, CO, USA)
OLIG2	Rabbit	1:200	GeneTex GTX132732 (Irvine, CA, USA)
GFAP	Rabbit	1:500	Novus NB300-141
HNA	Mouse	1:1000	Abcam ab191181 (Waltham, MA, USA)
MS-488	-	1:1000	Invitrogen A11029 (Waltham, MA, USA)
RB-594	-	1:200–750	Invitrogen A11037
DAPI prolong gold	-	-	Invitrogen P36935

## Data Availability

The original contributions presented in the study are included in the article/supplementary materials, further inquiries can be directed to the corresponding authors.

## Supplementary Materials

The following supporting information can be downloaded at https://www.mdpi.com/article/10.3390/biomedicines12081663/s1, Table S1. Description of mRS Scoring; Table S2. Piglet Mortality Reports.
