# Supplementary material for: Human-Induced Pluripotent Stem Cell-Derived Neural Stem Cell Therapy Limits Tissue Damage and Promotes Tissue Regeneration and Functional Recovery in a Pediatric Piglet Traumatic-Brain-Injury Model
Source: Biomedicines. 2024 Jul 25;12(8):1663. doi: 10.3390/biomedicines12081663 (PMC11351842; doi:10.3390/biomedicines12081663)
Supplement: Supplementary file 1 [file biomedicines-12-01663-s001.zip › biomedicines-3092534-supplementary.pdf]

		mRS Score
**Deceased**	Yes	**6**
	No	**0-5**
**General symptoms**	No symptoms	**0**
	No significant disability	**1**
	Requiring consistent care and attention	**2-5**
**Food/Drink**	Eating without assistance	**0-2**
	Drinking without assistance	**0-2**
	Eating with assistance	**3**
	Drinking with assistance	**3**
	Not eating or drinking	**5**
	Facial paralysis	**4**
**Motor function**	Walking without assistance	**0-3**
	Standing without assistance	**0-3**
	Walking with assistance	**4**
	Standing with assistance	**4**
	Circling to the right	**4**
	Circling to the left	**4**
	Unable to stand and move on own	**5**
**Bodily function**	Urination	**0-3**
	Defecation	**0-3**
**Level of consciousness**	Awake	**0-4**
	Asleep	**0-4**
	Sedated	**5**
	Requiring constant care and attention	**5**

Table S1. Description of mRS Scoring

Pig ID	Sex	Tx Group	Date of TBI	Date of Transplant	Date of Euthanasia	Necropsy results
27-23	F	PBS	10/20/2021	10/25/2021	10/29/2021	Necropsy not performed
17-4	F	PBS	3/9/2022	3/14/2022	4/13/2022	The TBI lesion is severe and probably communicates with the ventricle via the tract that had formed. Although the area of TBI looked potentially infected grossly and had abundant neutrophils microscopically, no bacteria were seen in the routinely stained sections. Severe tissue damage alone may have caused the severe inflammatory response. It is not clear exactly what triggered the more acute edema that lead to the cerebellar vermis herniation. Lesion progressive and various associated factors were likely responsible for the edema. The changes in the stomach do not appear to be associated with an infectious agent. Given the severe brain changes, endogenous steroids (stress) is likely at fault.
40-4	M	iNSC	5/4/2022	5/9/2022	5/12/2022	Cerebrum: Severe, chronic, focally extensive, suppurative of meningoencephalitis and ventriculitis with tract from cerebral surface into lateral ventricle with Gram-positive cocci in chains consistent with Streptococcus suis.<br>Cerebellum: Severe, chronic suppurative ventriculitis.<br>The changes in the brain are consistent with the isolation of Streptococcus suis. Histologically there was no evidence of pneumonia.
40-8	M	PBS	5/4/2022	5/9/2022	5/23/2022	Cerebrum: Severe, chronic, focally extensive, suppurative meningoencephalitis and ventriculitis with tract from cerebral surface into lateral ventricle.<br>Cerebellum: Severe, chronic suppurative ventriculitis.<br>The inflammatory changes are consistent with the Streptococcus suis that was isolated. An Escherichia coli was also isolated and is likely a contaminant, but could be a co-infection.
71-1	F	PBS	6/1/2022	6/6/2022	6/10/2022	Necropsy not performed
64-2	F	PBS	6/1/2022	6/6/2022	7/19/2022	Gross diagnosis: Extensive ulceration with hemorrhage of the pars esophagea, gastric perforation, diagphragmatic rent with mild herniation, bicavitary effusion with digesta, and Cerebral-dural adhesion, left side.<br>The primary problem was extensive ulceration of the pars esophagea with perforation at the edge of the region. This released digesta into the left side of the thorax through a rent in the diaphragm and into the abdomen. This resulted in bicavitary effusion. Gastric ulcers are a common problem in pigs and these animals have a number of potential causes- stress, feed, non-steroidal anti-inflammatory drugs (NSAIDs). Additionally, there was adhesion of the cerebral trauma site to the dura. It is unclear if this was a problem but it would interfere with cerebral spinal fluid (CSF) flow and could potentially result in healing with fibrosis in the parenchyma.

Table S2. Piglet Mortality Reports
